# The impacts of polyploidy, geographic and ecological isolations on the diversification of *Panax* (Araliaceae)

**DOI:** 10.1186/s12870-015-0669-0

**Published:** 2015-12-21

**Authors:** Feng-Xue Shi, Ming-Rui Li, Ya-Ling Li, Peng Jiang, Cui Zhang, Yue-Zhi Pan, Bao Liu, Hong-Xing Xiao, Lin-Feng Li

**Affiliations:** Key Laboratory of Molecular Epigenetics of the Ministry of Education (MOE), Northeast Normal University, Changchun, 130024 China; Institute of Grassland Science, Northeast Normal University, Key Laboratory of Vegetation Ecology, Ministry of Education, Changchun, 130024 China; Kunming Institute of Botany, Chinese Academy of Sciences, Kunming, 650201 China

**Keywords:** Chloroplast, Disjunt distribution, nrITS, Nucleotide diversity, *Panax*, Single copy nuclear gene, Whole genome duplication

## Abstract

**Background:**

*Panax* L. is a medicinally important genus within family Araliaceae, where almost all species are of cultural significance for traditional Chinese medicine. Previous studies suggested two independent origins of the East Asia and North America disjunct distribution of this genus and multiple rounds of whole genome duplications (WGDs) might have occurred during the evolutionary process.

**Results:**

We employed multiple chloroplast and nuclear markers to investigate the evolution and diversification of *Panax*. Our phylogenetic analyses confirmed previous observations of the independent origins of disjunct distribution and both ancient and recent WGDs have occurred within *Panax*. The estimations of divergence time implied that the ancient WGD might have occurred before the establishment of *Panax*. Thereafter, at least two independent recent WGD events have occurred within *Panax*, one of which has led to the formation of three geographically isolated tetraploid species *P. ginseng, P. japonicus* and *P. quinquefolius*. Population genetic analyses showed that the diploid species *P. notoginseng* harbored significantly lower nucleotide diversity than those of the two tetraploid species *P. ginseng* and *P. quinquefolius* and the three species showed distinct nucleotide variation patterns at exon regions.

**Conclusion:**

Our findings based on the phylogenetic and population genetic analyses, coupled with the species distribution patterns of *Panax*, suggested that the two rounds of WGD along with the geographic and ecological isolations might have together contributed to the evolution and diversification of this genus.

**Electronic supplementary material:**

The online version of this article (doi:10.1186/s12870-015-0669-0) contains supplementary material, which is available to authorized users.

## Background

Whole genome duplication (WGD) or polyploidy is thought to be central to the diversification of angiosperm plants [1, 2]. It is well recognized that all angiosperms are paleopolyploid [3, 4] and have experienced multiple rounds of WGD [5]. To date, about 30–70 % of the extant plant species are polyploidy [6]. The allopolyploid species reunite two or more sets of distinct genomes that entail a suite of genomic accommodations [7–9] which give rising to a variety of novel morphological and physiological phenotypes [10–12]. These observations have led to the hypothesis that polyploidy contributes to the diversification of angiosperm plants. Indeed, it has been demonstrated that 15 % of angiosperm speciation events are accompanied by ploidy increase [13]. In the grass tribe, for example, although no directly evidence indicates that the species diversification was accelerated by the allopolyploidy, at least one third of speciation events are associated with genetic allopolyploidy [14]. In addition, a series of studies from diverse plant taxa have documented that the “genomic shock” resulted from polyploidization has profound effects on the genetic architecture (e.g., gene loss), epigenetic modification (e.g., cytosine methylation) and gene expression (e.g., homeolog biased expression) [15–19], and some of these induced changes are linked to the phenotypic changes [20–22]. These attributes together suggested that polyploidy itself, as a mode of speciation and an avenue that generating novel variations, has indeed contributed to the evolution and diversification of plants.

*Panax* L. (Araliaceae) is a medicinally important genus in the East Asia and almost every species within the genus has cultural significance for traditional Chinese medicine [23]. The taxonomy of *Panax* has been controversial due to the circumscription of *P. pseudoginseng* and *P. japonicus* [24–26]. For example, all of the species from southwestern China have been treated as the varieties of *P. pseudoginseng* [27]. However, Zhou et al. [25] moved some *P. pseudoginseng* varieties (e.g., *P. pseudoginseng* var. *bipinnatifidus*) into the species *P. japonicus* based on their triterpenoids and seed morphologies. Thereafter, Wen and colleagues have reconstructed the phylogenetic trees of *Panax* based on nrITS and selected chloroplast genes [23, 28–30]. To date, seven well-recognized species and one species complex are defined according to their geographic distributions, chromosome numbers and phylogenetic relationships [30]. Based on the phylogenetic tree and chromosome number, Yi et al. [31] have proposed that at least two recent polyploidy events have occurred within the genus *Panax*, one of which has led to the formation of three geographically isolated tetraploid (2*n* = 48) species *P. ginseng*, *P. japonicus* and *P. quinquefolius*. The other recent polyploidy event had occurred within the *P. bipinnatifidus* species complex wherein both diploids (2*n* = 24) and tetraploids are identified. These previous studies provide a framework for understanding the evolutionary history of genus *Panax*. However, these phylogenetic analyses are mainly based on nrITS and selected chloroplast genes, the relationships and nucleotide variation patterns of diploid and tetraploid species remained uninvestigated. In addition, the fluorescence *in situ* hybridization (FISH) and genomic *in situ* hybridization (GISH) analyses revealed the allotetraploid of *P. ginseng* [32, 33]. More importantly, recent investigations based on the expressed sequence tags (ESTs) suggested that the tetraploid species *P. ginseng* and *P. quinquefolius* have experienced two rounds of WGD and diverged to each other after the recent tetraploidization event [34, 35]. These features suggested that the evolutionary trajectories of *Panax* species are much more complicated than we thought.

In this study, we employed 12 chloroplast genomes of *Panax* and relative genera to address if the ancient WGD has occurred before the establishment of genus *Panax*. To further infer the evolutionary trajectories of the extant *Panax* species, we applied nrITS, four chloroplast and seven single copy nuclear genes to investigate the phylogenetic relationships of the diploid and tetraploid species. To evaluate the impacts of polyploidization on the genetic diversity, we investigated the nucleotide variation pattern of two tetraploids, *P. ginseng* and *P. quinquefolius*, and one diploid species *P. notoginseng* based on 36 single copy nuclear genes. In comparison with the other congeneric species, the three economically important species are well recognized and cultivated widely in East Asia. The tetraploid species *P. ginseng* and *P. quinquefolius* have been used as a tonic and fatigue-resistance medicine in East Asia for a long time. Likewise, the diploid species *P. notoginseng* is considered to be a remedy for preventing bleeding and recovering from injury for thousands of years [30]. We expect our study shed lights on how the polyploidization, geographic and ecological isolations contribute to the evolution and diversification of genus *Panax*.

## Results

### Phylogenetic analyses of *panax*

**Fig. 1:**
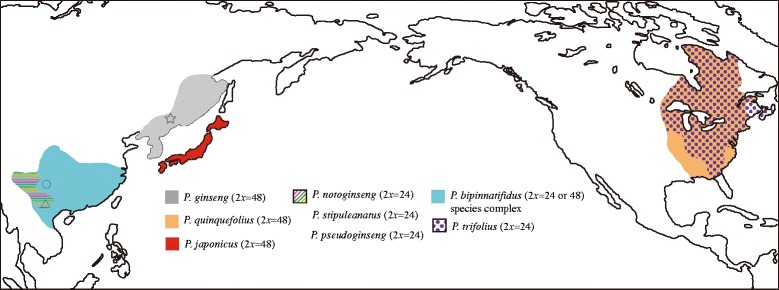
Geographic distributions and chromosome numbers of the extant diploid and tetraploid *Panax* species. The diploid species *P. trifolius* shows overlapped distribution with the tetraploid *P. quinquefolius*. The *P. bipinnatifidus* species complex covers the distribution ranges of *P. notoginseng*, *P. stipuleanatus* and *P. pseudoginseng*. Star, sampling locations of the species *P. ginseng* and *P. quinquefolius* in Jilin province of China; Circle, sampling location of *P. bipinnatifidus* species complex in Sichuan province of China; Triangle, sampling locations of *P. notoginseng*, *P. stipuleanatus* and *P. bipinnatifidus* species complex in Yunnan province of China. The original map was downloaded from Wikimedia Commons(https://commons.wikimedia.org/wiki/File:Map_of_the_Pacific_region.svg?uselang=zh-cn). The information of geographic distributions of *Panax* species was retrieved from the Natural Resources Conservation Service of USDA (www.plants.usda.gov) and Flora of China (www.efloras.org)

The geographic distributions and chromosome numbers of the *Panax* species are shown in Fig. 1. The lengths and informative characters of each alignment and detailed information of the specimens were presented in Additional file 1: Table S1 and Additional file 2: Table S2. In brief, the combined matrix of the four chloroplast genes includes 3031 characters, of which 211 (7.0 %) are variable sites. Similarly, the alignment of whole chloroplast genome of the 12 species contains a total of 144,303 bp in length and 11,506 (8.0 %) of which are polymorphic sites. In contrast, the percentages of informative characters in nrITS and single copy nuclear genes are apparently higher than those of chloroplast genes, which ranged from 8.2 % in *Z8* to 25.6 % in nrITS (Additional file 2: Table S2). The numbers of haplotype of the seven nuclear genes were shown in Additional file 2: Table S2 and accession numbers of the DNA sequences downloaded from GenBank were listed in Additional file 3: Table S3.

**Fig. 2:**
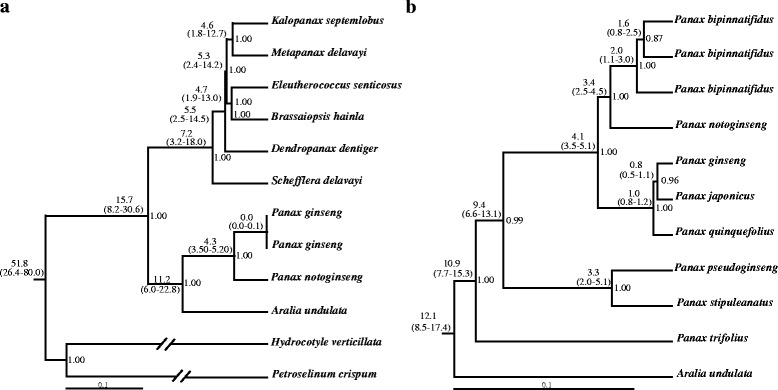
Divergence times and topologies of Bayesian trees based on whole chloroplast genome (**a**) and four selected chloroplast genes (**b**). The values on the left and right of each node are the divergence time (one million years ago) and poster prior support, respectively. Length of each branch is not shown in the two phylogenetic trees

**Fig. 3:**
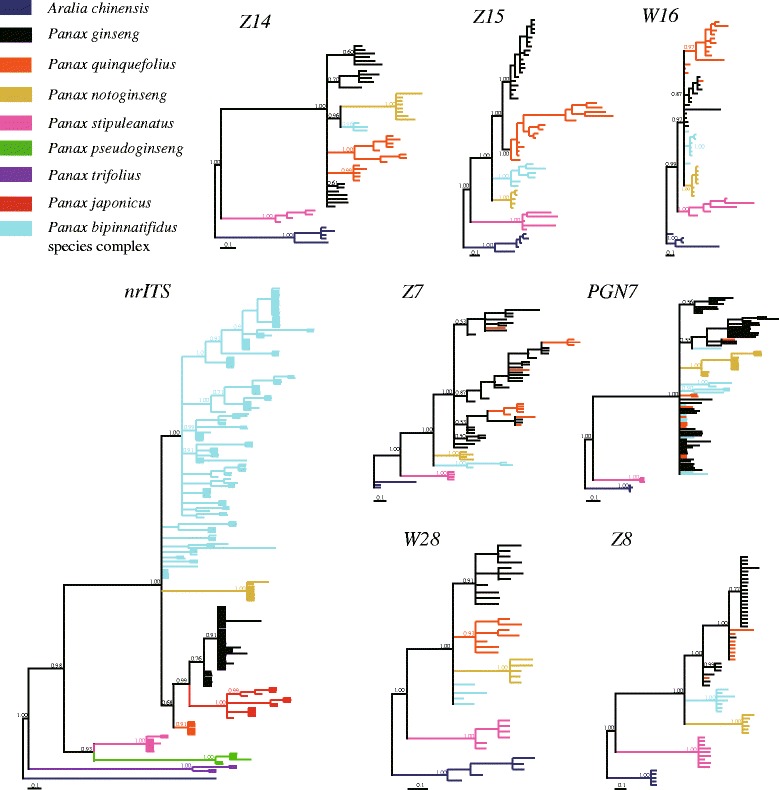
Topologies of Bayesian trees based on nrITS and seven single copy nuclear genes. Each branch represents one haplotype and identical sequences from the same species were removed. The color of branch stands for different species. Numbers of haplotypes for each gene were shown in Additional file 2: Table S2

Phylogenetic reconstruction using Bayesian inference (BI) resulted in distinct topologies between the chloroplast and nuclear datasets (Fig. 2 and 3). In detail, the BI tree based on whole chloroplast genome revealed that species of genera *Aralia* and *Panax* grouped together as a clade, supporting previous observation that the two lineages are the closest genera within family Araliaceae (Fig. 2a). To this end, we employed the *Aralia* species as outgroup when we performed the phylogenetic analyses of the *Panax* species. As shown in the Fig. 2b, the North American diploid species *P. trifolius* was placed at the basal clade with high support value (poster prior value = 1.00). Likewise, the two Asiatic diploid species *P. pseudoginseng* and *P. stipuleanatus* formed a monophyletic clade and showed distinct phylogenetic positions to the other Asiatic species. It should be noted that the remaining species were separated into two distinct lineages, one of which contains the three tetraploid species *P. ginseng*, *P. japonicus* and *P. quinquefolius*, and the other clade includes the *P. notoginseng* and *P. bipinnatifidus* species complex. These features suggested that the two lineages shared the ancestral chloroplast genome and differed from the three basal diploid species.

In contrast, the BI trees of nrITS and seven nuclear genes revealed more complicated phylogenetic topologies for the *Panax* species (Fig. 3). For example, although the phylogenetic positions of the three basal species, *P. trifolius*, *P. stipuleanatus* and *P. pseudoginseng*, showed no significant differences between chloroplast and nrITS topologies, accessions of the *P. bipinnatifidus* species complex were not clustered together with *P. notoginseng* as a monophyletic clade in the BI tree of nrITS (Fig. 3). Instead, they exhibited polyphyletic pattern and then grouped together with the *P. notoginseng* and three tetraploid species *P. ginseng*, *P. japonicus* and *P. quinquefolius*. The *P. bipinnatifidus* accessions used in this study contains both diploids and tetraploids and cover their current distributions from southeastern and southwestern China. The polyphyletic pattern suggested the possibility of heterogeneous origins of this species complex. Similarly, topologies of the seven nuclear genes also revealed that *P. stipuleanatus* showed a distinct phylogenetic position to the other Asiatic species (Fig. 3). These findings suggested that the *P. notoginseng* and *P. bipinnatifidus* species complex are more close to the tetraploid species *P. ginseng* and *P. quinquefolius* than those of the basal diploid species (Fig. 3). However, we noted that the nrITS topology showed an autotetraploid pattern of the three tetraploid species. In contrast, topologies of the seven nuclear genes revealed that the haplotypes of *P. ginseng* and *P. quinquefolius* mixed together at most of these nuclear genes, clearly supporting the allotetraploid of the two species. Taken together, our results based on chloroplast and nuclear genes indicated that *P. ginseng* and *P. quinquefolius* are allotetraploid and all accessions of the *P. bipinnatifidus* species complex have the same maternal origin.

### Whole genome duplication and divergence time

Previous investigations based on expression sequence tags (ESTs) have documented that the tetraploid (2*n* = 48) species *P. ginseng* and *P. quinquefolius* have experienced two rounds of WGD [34, 36]. To this end, we estimated the divergence times of the *Panax* species based on four chloroplast genes and whole chloroplast genome, respectively. Estimations of the divergence time showed that the genus *Panax* diverged from *Aralia* some 11.2 million years ago (MYA) (95 % confidence interval (CI): 6.0–22.8 MYA) for whole chloroplast genome data (Fig. 2a) and 12.1 MYA (CI: 8.5–17.4 MYA) for the four chloroplast genes (Fig. 2b), respectively. Thereafter, the basal species *P. trifolius* and the ancestor of *P. stipuleanatus* and *P. pseudoginseng* diverged from the remaining species before 9.4 MYA (CI: 6.6–13.1 MYA) (Fig. 2b). Notably, our results revealed that the three tetraploid species, *P. ginseng*, *P. japonicus* and *P. quinquefolius*, shared the same maternal donor and diverged to each other during 0.8–1.0 MYA (CI: 0.5–1.2 MYA) (Fig. 2b). In contrast, the divergence time between *P. notoginseng* and *P. bipinnatifidus* species complex is earlier than those of the three tetraploid species. It was suggested that the *P. bipinnatifidus* species complex have also experienced recent WGD [31]. In our study, the exact origins of the *P. bipinnatifidus* species complex can not be determined due to the limited sampling size used at the single copy nuclear genes and undetermined chromosome numbers. However, our phylogenetic results showed that the Asiatic diploid species *P. notoginseng* shared the maternal genome with the *P. bipinnatifidus* species complex (Fig. 2b) but showed independent phylogenetic position at the nrITS and nuclear genes (Fig. 3). Likewise, the Asiatic diploid species *P. stipuleanatus* was placed at the basal clade at the chloroplast, nrITS and nuclear genes, suggesting that it was not involved in the two recent WGD events. Similar phenomenon was also found in the two North American species where demonstrated that although both of the diploid *P. trifolius* and tetraploid *P. quinquefolius* are distributed in the North America, the two species fall into two distinct clades (Fig. 2b and 3).

### Nucleotide diversity

**Fig. 4:**
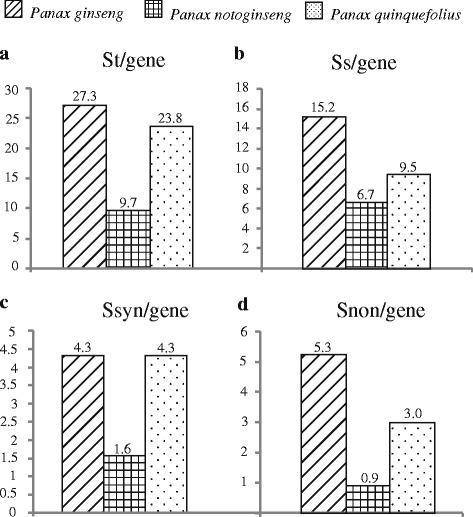
Quantification of segregating site per gene at total **a**, species-specific **b** synonymous **c** and nonsynonymous **d** sites for the 36 genes. The numbers above the vertical bars are the exact numbers of segregating sites per gene. Detailed information of the 36 nuclear genes was shown in Additional file 4: Table S4

To estimate if the orthologs showed heterogeneous evolutionary rates among the diploid and tetraploid species, we compared the nucleotide variation pattern of *P. notoginseng*, *P. ginseng* and *P. quinquefolius* based on 36 single copy nuclear genes. As shown in our results, the diploid species *P. notoginseng* harbored significantly lesser number of variations at total (St), synonymous (Ssyn) and nonsynonymous (Snon) sites than those of the two tetraploid species *P. ginseng* and *P. quinquefolius* (Fig. 4 and Additional file 4: Table S4, *t*-test, all *p* values < 0.003). For instance, the St of *P. notoginseng* ranged from 0 (locus *W13*, *W31* and *W59*) to 58 (locus *W48*), while the St varied from 5 (locus *W28* and *W31*) to 92 (locus *Z63*) and 4 (locus *W28*) to 94 (locus *Z63*) in *P. ginseng* and *P. quinquefolius*, respectively (Additional file 4: Table S4). Similar results were also observed at the parameter π_T_ where most of the 36 genes showed obviously lower nucleotide diversity in *P. notoginseng* than those of *P. ginseng* and *P. quinquefolius* (Additional file 4: Table S4). In particular, we noted that the decreasing of nucleotide diversity at exon regions of the 36 nuclear genes is more apparent than that of the intron regions (Additional file 4: Table S4). For example, ten of the 36 nuclear genes in *P. notoginseng* showed no variations at the exon regions, but both synonymous and nonsynonymous muations were reported in the *P. ginseng* and *P. quinquefolius*. In addition, the *P. notoginseng* also showed significantly lower ka/ks values compared to the tetraploid species *P. ginseng* and *P. quinquefolius* (Additional file 4: Table S4, *t*-test, both *p* values < 0.03).

To further evaluate the impacts of tetraploidization on the genetic constitution of tetraploid species, we compared the nucleotide variation pattern between the two tetraploids *P. ginseng* and *P. quinquefolius*. Our results revealed that the Asiatic tetraploid *P. ginseng* harbored slightly greater number of St than that of the North America tetraploid *P. quinquefolius* (Fig. 4 and Additional file 4: Table S4, *t*-test, *p* = 0.364). Notably, the two tetraploid species exhibited distinct nucleotide variation pattern at the exon regions (Fig. 4 and Additional file 4: Table S4). For instance, most of the 36 nuclear genes showed higher nonsynonymous mutation rates in the *P. ginseng* compared to *P. quinquefolius* (Fig. 4 and Additional file 4: Table S4, *t*-test, *p* = 0.02). Similarly, ka/ks values of the 36 nuclear genes also exhibited obviously different between *P. ginseng* and *P. quinquefolius* (Additional file 4: Table S4). It should be noted that each of the three species possessed high level of species-specific SNPs (Fig. 4 and Additional file 4: Table S4). For example, although the two tetraploid *P. ginseng* and *P. quinquefolius* diverged recently, 495 and 313 SNPs are specific to each of the two tetraploids.

## Discussion

### Ancient and recent polyploidy followed by geographic and ecological isolations

Polyploidy is a widespread feature of plant genomes and has played a crucial role in the evolution and diversification of plants [37]. In terms of time of origin, polyploidy can be broadly divided into paleopolyploidization (ancient WGD) and neopolyploidization (recent WGD) [38]. The recent polyploidy events are easily identified by the chromosome numbers, genome size, and gene copy number relative to progenitors. In contrast, the evidence of ancient polyploidy has mainly come from comparative genetic mapping, analysis of specific gene families or by the identification of duplicated genes in ESTs [39].

The contributions of ancient WGD on the evolution and diversification of plants are well-recognized [40–42]. In the case of legumes, for example, multiple independent polyploidy events had occurred in the early radiation stage and which might provide raw materials for the genetic innovations that resulted in the evolution of symbiotic nitrogen fixation [43–45]. In *Panax*, previous studies based on the ESTs indicated that the extant tetraploid species, *P. ginseng* and *P. quinquefolius*, have undergone two rounds of WGD, of which, the first round of WGD had occurred during 24.6–32.8 MYA [34, 35]. Here, our results based on phylogenetic and divergence time analyses suggested that the genus *Panax* have experienced both ancient and recent WGDs. Given that both of genera *Panax* and *Aralia* have the same basic chromosome number (*n* = 12) [31] and diverged to each other obviously later than that of the first round WGD, we proposed that the ancient WGD might have occurred before of the establishment of genus *Panax*. Under this hypothesis, it is tempting to predict that the extant diploid species of *Panax* are paleopolyploid, which is thought to be a common phenomenon in plants [46]. We noted that the genome sizes of extant diploid and tetraploid *Panax* species vary dramatically [47–49]. Similar observations were also reported in *Gossypium* and *Arabidopsis* where rapid genomic revolution during and/or soon after WGD and gradual process of diploidization are likely to result in variation and evolution in genome size [50–53]. Taken together, our findings indicated that the ancient WGD might have contributed to the evolution and diversification of *Panax*. In addition to the ancient WGD, recent polyploidy events were also revealed by our phylogenetic and divergence time analyses. However, we noted that the nrITS topology did not show the allotetraploid of the three species *P. ginseng*, *P. japonicus* and *P. quinquefolius*, which is not consistent with previous observations based on FISH and GISH [33]. The possible explanation might be that the orthologs from distinct genomes were homogenized through concerted evolution. As expected, topologies of the seven single copy nuclear genes confirmed the allotetraploid of the three species. It should be noted that both the four selected chloroplast genes and nrITS topologies suggested the single tetraploidization origin of the three tetraploid species. Similar phenomenon was also reported in the *Gossypium* where five extant tetraploid species (AADD) have derived from a single polyploidization event between *G. raimondii* (DD) and two extant A-genome species about 1–2 MYA [54–57]. In our case, however, the diploid species used in this study may not the direct progenitors of the three tetraploid species. Instead, our phylogenetic results suggested a possibility that the three tetraploids have the same maternal donor and might share the parental ancestor with *P. notoginseng* and *P. bipinnatifidus* specie complex. Similar phylogenetic patterns were also observed in the *Panicum* where the two teraploids *P. miliaceum* and *P. repens* shared the same parental genome but have distinct maternal donors [58]. To this end, it is possible that the direct donors of the three tetraploid species may not exist at present. It has also been suggested that recent WGD had occurred within the *P. bipinnatifidus* species complex [31]. In our study, although the diploid species *P. notoginseng* showed overlapped distributions with the *P. bipinnatifidus* species complex, phylogenetic analyses indicated that it might not be involved in the recent WGD of *P. bipinnatifidus* species complex. Instead, the observed polyphyletic pattern suggested that polyploids within the *P. bipinnatifidus* species complex might have formed through autopolyploidization. Together, our findings suggested that the recent WGDs have indeed promoted the diversification of *Panax*.

It has been demonstrated that the genus *Panax* shows a disjunct distribution between eastern Asia and eastern North America [59–61]. Here, our results confirmed previous hypothesis of two independent origins of the disjunct distributions of *Panax* [23, 28]. In particular, we noted that the diploid species *P. trifolius* was not involved in the tetraploidization of *P. quinquefolius*, although they showed overlapped distribution pattern in the eastern North America. In contrast, despite the three geographic isolated tetraploids, *P. ginseng*, *P. quinquefolius* and *P. japonicus*, are endemic to northeastern Asia (excluding Japan), North America and Japan, respectively, they have established through a single tetraploidization event and diverged almost simultaneous (0.5–1.2 MYA). These features suggested that geographic isolation is likely one of the underlying mechanisms that promoted the divergence of the three tetraploids. In addition, it has been reported that, in the *P. bipinnatifidus* species complex, tetraploids usually occur at high altitudes [28, 29]. Similar observations were also reported in the *Alyssum montanum*-*A. repens* complex in which the polyploidy provides raw materials for diversification and the geographic and ecological isolation have further stimulated speciation [62]. Under this hypothesis, our findings suggested that multiple rounds of ancient and recent polyploidization, along with geographic and ecological isolations, might have together played important roles in the evolution and diversification of *Panax*.

### Nucleotide diversity of diploid and tetraploid species

It was widely recognized that WGD has profound effects on the genome constitution of plants [8, 11, 15, 17, 19, 63]. The notable feature of polyploidy is that it would increase the copy numbers of a given gene. As a result, orthologs in the polyploids would harbor relatively higher genetic diversity and heterozygosity compared to the diploids, mainly due to the relaxed selection and reuniting of multiple parental copies [16, 64–67]. In the case of *Gossypium*, for example, the population genetic analyses based on 48 nuclear genes showed that polyploidy in *Gossypium* has led to a modest enhancement in rates of nucleotide substitution [68]. Here, our study also demonstrated that the two tetraploid species, *P. ginseng* and *P. quinquefolius*, showed relatively higher nucleotide diversity at the total sites of the 36 nuclear genes than those of the diploid species *P. notoginseng*. The possible explanation might be that the two allotetraploids possessed two divergent genomes which would increase the heterozygous and nucleotide diversity at the genome-wide level. In addition, the limited sampling size of *P. notoginseng* might be also respossible for the low nucleotide diversity. Notably, we found that a vast of majority of SNPs is specific to each of the three species, suggesting that some of these SNPs might have accumulated after their divergent. Given the recent divergence and allopatric distributions of the three species, we propose that, in addition to the effects of recent WGD, geographic isolation might have also contributed to the distinct variation patterns of the three species.

Previous studies have suggested that gene duplication plays a crucial role in the coding sequence evolution [64, 69–71]. In the hexaploid wheat, duplicated orthologs that created by WGD can change the dynamic of coding sequence evolution through relaxing selection and then provide chances for the accumulation of new mutations which may impact gene function [72]. In our study, we found that, compared to the introns of the 36 nuclear genes, the deceasing in nucleotide diversity at exons in diploid species is more apparent than those of the two tetraploid species. In particular, the diploid species showed obviously lower ka/ks values at the 36 nuclear genes. In addition, we also noted that distinct variation pattern was also observed between the two tetraploid species. Taken the locus *Z8* as an example, only two synonymous mutations were found in *P. quinquefolius*, yet eight and five synonymous and nonsynonymous mutations were identified in *P. ginseng*. These findings allow us to speculate that gene duplication might provide raw materials and natural selection favors different mutations between the diploid and tetraploid species.

## Conclusion

WGD is thought to be a driving force that promoted the evolution and diversification of plants. Here, our phylogenetic analyses based on multiple chloroplast and nuclear genome markers demonstrated that the ancient and recent WGDs along with geographic and ecological isolations have together contributed to the diversification of *Panax* species. Through comparing the nucleotide variation patterns of the diploid and tetraploid species, we found that distinct selection pressures might have acted on these nuclear genes during their evolutionary processes.

## Methods

### Sampling and DNA extraction

The aims of this study are to infer the phylogenetic relationships of the extant *Panax* species and evaluate if the same ortholog exhibits heterogeneous evolutionary rates between the diploid and tetraploid species. To this end, 11 and 15 individuals of *P. notoginseng* (Burkill) Chen ex and *P. ginseng* were collected from the Yunnan and Jilin provinces of China, respectively. Similarly, seven and eight accessions of *P. quinquefolius* L. and *P. stipuleanatus* Tsai and Feng were collected from the Jilin and Yunnan provinces of China, respectively. Samples of these species were collected from a wide geographic area that several populations were included. In addition, four accessions sampled from Yunnan and Sichuan provinces of China were chosen to represent the *P. bipinnatifidus* Seem. species complex. The exact geographic locations of these samples were shown in Fig. 1. The four species, *P. bipinnatifidus*, *P. stipuleanatus P. ginseng* and *P. notoginseng*, are widely distributed in southwestern and northeastern China and no specific permissions are required for the specimen collection. The species *P. quinquefolius* is naturally distributed in North America and widely cultivated in North America and northeastern China. We collected seven cultivated accessions of *P. quinquefolius* from Jilin province of China with the owner’s permission. The remaining 13 accessions of *P. quinquefolius* were obtained from our collaborator who bought these samples from the market of the United States of America. The exact geographic location of these accessions is unclear. Detailed information of the specimens used in this study is listed in Additional file 1: Table S1. Genomic DNA was extracted from the silica-gel dried leaf material of each accession using Qiagen (Tiangen, Beijing) following the manufacturer’s instructions.

### Chloroplast, nrITS and single copy nuclear gene selection

To infer the establishment and evolutionary process of *Panax*, we downloaded the whole chloroplast genomes of two *Panax* and nine relative genera species from GenBank (*Panax ginseng*, KF431956 and KC686332; *Panax notoginseng*, KJ566590; *Aralia undulata*, KC456163; *Dendropanax dentiger*, KP271241; *Metapanax delavayi*, KC456165; *Kalopanax septemlobus*, KC456167; *Eleutherococcus senticosus*, JN637765; *Brassaiopsis hainla*, KC456164; *Schefflera delavayi*, KC456166; *Hydrocotyle verticillata*, HM596070; *Petroselinum crispum*, HM596073). To further address the evolutionary trajectories of the extant diploid and tetraploid *Panax* species, we employed the nrITS and four chloroplast genes (*trnD*, *psbK-psbI, rbcL* and *ycf1*) to reconstruct the phylogenetic trees (Additional file 2: Table S2 and Additional file 3: TableS 3). The nrITS region is one of the most popular nuclear DNA regions in molecular phylogenetic studies, yet the intra-individual paralogy [73–75] and concerted evolution have largely limited its application in the phylogenetic work, especially in the polyploid species. Instead, single or low copy nuclear genes have been proposed to be particularly useful in resolving such problems and are an increasingly popular alternative to nrITS [76]. To this end, 53 single copy nuclear genes were selected according to our previous studies [77, 78], 36 of which were successfully amplified in the diploid species *P. notoginseng* and tetraploid species *P. ginseng and P. quinquefolius* (Additional file 5: Table S5). Seven genes that showed high transferability across the genera *Panax* and *Aralia* were used to construct the phylogenetic trees (Additional file 2: Table S2).

### PCR, sequencing and phylogenetic analyses

Polymerase chain reactions (PCRs) of the single copy nuclear genes were performed in a 50 μL volume containing 0.2 mM of each dNTP, 1.5 mM MgCl_2_, 0.5 mM of each primer, 1U of rTaq polymerase (Takara, Dalian, China), and about 50 ng of DNA template under the following conditions: 5 min at 95 °C, followed by 30 cycles of 30 s at 94 °C, 30 s at the annealing temperature of each primer combination (Additional file 5: Table S5), 60 s at 72 °C, and then a final 5 min extension at 72 °C. The amplifications of seven single copy nuclear genes were purified with Gel Band Purification Kit (Tiangen, Beijing, China) and cloned using pMD18 vector (Takara, Dalian, Liaoning) following the manufacturer’s instructions. To obtain different haplotypes of the seven nuclear genes, multiple accessions of each species were selected and 4–10 clones were sequenced for each accession studied.

The DNA sequences were aligned using the default parameters in Clustal [79] and edited manually using BioEdit [80] if necessary. To infer the phylogenetic relationships of the *Panax* species, the BI analyses for the nrITS, combined chloroplast and single copy nuclear genes were performed using MrBayes [81], separately. Model parameters for each data set were estimated using jModelTest [82]. The best-fit models for each data set were showed in Additional file 2: Table S2. For the Bayesian trees, two independent Markov chains were run and calculated simultaneously with 1,000,000 generations for each data set. The convergence of the two runs was evaluated by stopping the analysis when the average standard deviation was below 0.01. Bayesian posterior probabilities were estimated as the majority consensus of all sampled trees with the first 25 % discarded as burn-in. The divergence times of *Panax* and relative genera were calculated using mcmctree of PAML [83, 84]. The indepented rates and HKY85 were chosen as the molecular clock and nucleotide substitution model, respectively. The ambiguity characters were removed from alignments. The empirical divergence times of *P. ginseng*/*P. quinquefolius* (0.8–1.2 MYA) and *P. ginseng*/*P. notoginseng* (3.5–5.2 MYA) [35, 36] were assigned to constrain the age of the *Panax*. A Birth-Death prior on branching rates was employed and three independent analyses were run for 10,000 generations.

### SNP recalibrating and nucleotide diversity

The references of the 36 single copy nuclear genes were obtained from our previous studies [77, 78]. The population data of *P. ginseng*, *P. quinquefolius* and *P. notoginseng* were sequenced using Illumina Hiseq 2000 (BGI, Shenzhen, China). The quality of raw reads was checked using FastQC [85] and low-quality (Phred < 30) reads were removed. Alignments of the clean reads were initially screened against the obtained references using Burrows-Wheeler Aligner [86]. The low quality single nucleotide polymorphisms (SNPs) (mapping quality < 30, depth < 10) and PCR duplicates were removed from the mapped reads using SAMtools [87]. The heterozygous and homozygous SNPs were reported according to our previous study [78]. The Perl scripts were applied to generate the alignment for each gene by replacing the references with reported SNPs. Insertions/deletions (INDELs) were excluded from the subsequent data analyses. Accordingly, a total of 0.55 million 100 bp paired-end reads (low quality reads and PCR duplicates were removed) were mapped to the references. We therefore obtained an average of ~80.6 × coverage for each gene per individual. The numbers of species-specific SNPs for each species were estimated based on the total segregating sites of the three species. The nucleotide diversity of the three *Panax* species was calculated using DnaSP v5 [88], including number of segregating sites (S), ration of nonsynonymous and synonymous site (Ka/Ks), nucleotide diversity π [89] for total, nonsynonymous and synonymous sites, respectively. The segregating sites that showed monomorphic within each of the three species were not included in the analyses of nucleotide diversity.

### Availability of supporting data

All data generated from this study were submitted to GenBank under the accession number KT593555-KT593862 and PRJNA291547.

## Additional files

Additional file 1: Table S1. Accessions of *Panax* species and outgroups sampled from the 36 single copy nuclear genes. (DOCX 43 kb) Additional file 2: Table S2. Detailed information of the chloroplast and nuclear genes used in this study. (DOCX 58 kb) Additional file 3: Table S3. Accession numbers of the *Panax* species and outgroups used in this study. (DOCX 23 kb) Additional file 4: Table S4. Nucleotide variation patterns of the 36 single copy nuclear genes used in this study. (DOCX 34 kb) Additional file 5: Table S5. Detailed information of the 36 single copy nuclear genes used in this study. (DOCX 22 kb)

## References

[1] Freeling M, Thomas BC (2006). Gene-balanced duplications, like tetraploidy, provide predictable drive to increase morphological complexity. Genome Res.

[2] Wall PK, Soltis PS, DePamphilis CW, Soltis DE, Albert VA, Leebens-Mack J (2009). Polyploidy and angiosperm diversification. Am J Bot.

[3] Bowers JE, Chapman BA, Rong J, Paterson AH (2003). Unravelling angiosperm genome evolution by phylogenetic analysis of chromosomal duplication events. Nature.

[4] Jiao Y, Wickett NJ, Ayyampalayam S, Chanderbali AS, Landherr L, Ralph PE (2011). Ancestral polyploidy in seed plants and angiosperms. Nature.

[5] Renny-Byfield S, Wendel JF (2014). Doubling down on genomes: polyploidy and crop plants. Am J Bot.

[6] Ramsey J, Schemske DW (1998). Pathways, mechanisms, and rates of polyploid formation in flowering plants. Annu Rev Ecol Syst..

[7] Osborn TC, Pires JC, Birchler JA, Auger DL, Chen ZJ, Lee H-S (2003). Understanding mechanisms of novel gene expression in polyploids. Trends Genet.

[8] Adams KL, Wendel JF (2005). Polyploidy and genome evolution in plants. Curr Opin Plant Biol.

[9] Chen ZJ (2007). Genetic and epigenetic mechanisms for gene expression and phenotypic variation in plant polyploids.

[10] Adams KL (2007). Evolution of duplicate gene expression in polyploid and hybrid plants. J Hered.

[11] Madlung A (2013). Polyploidy and its effect on evolutionary success: old questions revisited with new tools. Heredity.

[12] Otto SP, Whitton J (2000). Polyploid incidence and evolution. Annu Rev Genet.

[13] Wood TE, Takebayashi N, Barker MS, Mayrose I, Greenspoon PB, Rieseberg LH (2009). The frequency of polyploid speciation in vascular plants. Proc Natl Acad Sci U S A.

[14] Estep MC, McKain MR, Diaz DV, Zhong J, Hodge JG, Hodkinson TR (2014). Allopolyploidy, diversification, and the Miocene grassland expansion. Proc Natl Acad Sci U S A.

[15] Doyle JJ, Flagel LE, Paterson AH, Rapp RA, Soltis DE, Soltis PS (2008). Evolutionary genetics of genome merger and doubling in plants.

[16] Flagel LE, Wendel JF (2009). Gene duplication and evolutionary novelty in plants. New Phytol.

[17] Adams KL, Wendel JF, Chen ZJ, Birchler JA (2013). Dynamics of Duplicated Gene Expression in Polyploid Cotton. Polyploid and Hybrid Genomics.

[18] Jiao Y, Paterson AH (2014). Polyploidy-associated genome modifications during land plant evolution. Philos Trans R Soc Lond B Biol Sci.

[19] Soltis DE, Visger CJ, Soltis PS (2014). The polyploidy revolution then…and now: Stebbins revisited. Am J Bot.

[20] Pires JC, Zhao J, Schranz M, LEON EJ, Quijada PA, Lukens LN (2004). Flowering time divergence and genomic rearrangements in resynthesized *Brassica polyploids* (Brassicaceae). Biol J Linn Soc.

[21] Gaeta RT, Pires JC, Iniguez-Luy F, Leon E, Osborn TC (2007). Genomic changes in resynthesized *Brassica napus* and their effect on gene expression and phenotype. Plant Cell.

[22] Ni Z, Kim E-D, Ha M, Lackey E, Liu J, Zhang Y (2009). Altered circadian rhythms regulate growth vigour in hybrids and allopolyploids. Nature.

[23] Choi H-K, Wen J (2000). A phylogenetic analysis of *Panax* (Araliaceae): Integrating cpDNA restriction site and nuclear rDNA ITS sequence data. Plant Syst Evol.

[24] Hara H (1970). On the Asiatic species of the genus *Panax*. J Japanese botany.

[25] Zhou J, Huang W, Wu M, Yang C, Feng K, Wu Z (1975). Triterpenoids from *Panax* Linn. and their relationship with taxonomy and geographical distribution. Acta Phytotaxon Sin.

[26] Hoo G, Tseng Cj, Tsai SC. Flora reipublicae popularis Sinicae delectis florae reipublicae popularis *Sinicae agendae* academiae Sinicae edita: Tom 54. Angiospermae. Dicotyledoneae. Araliaceae. Facultas Biologica Universitatis Amoiensis; Beijing, 1978.

[27] Ho C, Tseng C (1973). On the Chinese species of *Panax Linn*. Acta Phytotaxonom Sinica.

[28] Wen J, Zimmer EA (1996). Phylogeny and biogeography of *Panax L*. (the ginseng genus, araliaceae): inferences from ITS sequences of nuclear ribosomal DNA. Mol Phylogenet Evol.

[29] Lee C, Wen J (2004). Phylogeny of *Panax* using chloroplast trnC-trnD intergenic region and the utility of *trnC-trnD* in interspecific studies of plants. Mol Phylogen Evol.

[30] Zuo Y, Chen Z, Kondo K, Funamoto T, Wen J, Zhou S (2011). DNA barcoding of *Panax* species. Planta Med.

[31] Yi T, Lowry PP, Plunkett GM (2004). Chromosomal evolution in Araliaceae and close relatives. Taxon.

[32] Choi HW, Koo DH, Bang KH, Paek KY, Seong NS, Bang JW (2009). FISH and GISH analysis of the genomic relationships among *Panax* species. Genes Genom.

[33] Choi HI, Waminal NE, Park HM, Kim NH, Choi BS, Park M (2014). Major repeat components covering one-third of the ginseng (*Panax ginseng* C.A. Meyer) genome and evidence for allotetraploidy. Plant J.

[34] Kim NH, Choi HI, Kim KH, Jang W, Yang TJ (2014). Evidence of genome duplication revealed by sequence analysis of multi-loci expressed sequence tag-simple sequence repeat bands in *Panax ginseng* Meyer. J Ginseng Res.

[35] Choi HI, Kim NH, Lee J, Choi BS, Do Kim K, Park JY (2013). Evolutionary relationship of *Panax ginseng* and *P. quinquefolius* inferred from sequencing and comparative analysis of expressed sequence tags. Genet Resour Crop Evol.

[36] Choi HI, Kim NH, Kim JH, Choi BS, Ahn I-O, Lee JS (2011). Development of reproducible EST-derived SSR markers and assessment of genetic diversity in *panax ginseng* cultivars and related species. J Ginseng Res.

[37] Soltis PS, Soltis DE. Polyploidy and genome evolution. New York: Springer; 2012. pp. 225-249.

[38] Hilu K (1993). Polyploidy and the evolution of domesticated plants. Am J Bot..

[39] Schranz ME, Mitchell-Olds T (2006). Independent ancient polyploidy events in the sister families Brassicaceae and Cleomaceae. Plant Cell.

[40] Paterson AH, Bowers JE, Chapman BA (2004). Ancient polyploidization predating divergence of the cereals, and its consequences for comparative genomics. Proc Natl Acad Sci U S A.

[41] Solds DE, Bell CD, Kim S, Soltis PS (2008). Origin and early evolution of angiosperms. Year in Evol Biol.

[42] Van de Peer Y, Maere S, Meyer A (2009). The evolutionary significance of ancient genome duplications. Nat Rev Genet.

[43] Young ND, Debellé F, Oldroyd GE, Geurts R, Cannon SB, Udvardi MK (2011). The Medicago genome provides insight into the evolution of rhizobial symbioses. Nature.

[44] Li QG, Zhang L, Li C, Dunwell JM, Zhang YM (2013). Comparative genomics suggests that an ancestral polyploidy event leads to enhanced root nodule symbiosis in the Papilionoideae. Mol Biol Evol.

[45] Cannon SB, McKain MR, Harkess A, Nelson MN, Dash S, Deyholos MK (2015). Multiple polyploidy events in the early radiation of nodulating and nonnodulating legumes. Mol Biol Evol.

[46] Tate JA, Joshi P, Soltis KA, Soltis PS, Soltis DE (2009). On the road to diploidization? Homoeolog loss in independently formed populations of the allopolyploid *Tragopogon miscellus* (Asteraceae). BMC Plant Biol.

[47] Pan YZ, Zhang YC, Gong X, Li FS (2014). Estimation of genome size of four *Panax* species by flow cytometry. Plant Diversity Res.

[48] Hong C, Lee S, Park J, Plaha P, Park Y, Lee Y (2004). Construction of a BAC library of Korean ginseng and initial analysis of BAC-end sequences. Mol Genet Genomics.

[49] Obae GS (2012). Nuclear DNA, content and genome size of American ginseng. J Med Plants Res..

[50] Cronn RC, Small RL, Haselkorn T, Wendel JF (2002). Rapid diversification of the cotton genus (*Gossypium*: Malvaceae) revealed by analysis of sixteen nuclear and chloroplast genes. Am J Bot.

[51] Paterson AH, Wendel JF, Gundlach H, Guo H, Jenkins J, Jin D (2012). Repeated polyploidization of Gossypium genomes and the evolution of spinnable cotton fibres. Nature.

[52] Hendrix B, Stewart JM (2005). Estimation of the nuclear DNA content of gossypium species. Ann Bot.

[53] Wolf DE, Steets JA, Houliston GJ, Takebayashi N (2014). Genome size variation and evolution in allotetraploid *Arabidopsis kamchatica* and its parents, *Arabidopsis lyrata* and *Arabidopsis halleri*.. AoB Plants..

[54] Wendel JF (1989). New world tetraploid cottons contain old world cytoplasm. Proc Natl Acad Sci U S A.

[55] Wendel JF, Cronn RC (2003). Polyploidy and the evolutionary history of cotton. Adv Agron.

[56] Grover CE, Grupp KK, Wanzek RJ, Wendel JF (2012). Assessing the monophyly of polyploid Gossypium species. Plant Syst Evol.

[57] Grover CE, Gallagher JP, Jareczek JJ, Page JT, Udall JA, Gore MA (2015). Re-evaluating the phylogeny of allopolyploid *Gossypium L*. Mol Phylogenet Evol.

[58] Hunt HV, Badakshi F, Romanova O, Howe CJ, Jones MK, Heslop-Harrison JS (2014). Reticulate evolution in *Panicum* (Poaceae): the origin of tetraploid broomcorn millet. P miliaceum J Exp Bot.

[59] Li HL (1952). Floristic relationships between eastern Asia and eastern North America. Trans Am Philos Soc..

[60] Zhengyi W (1983). On the significance of Pacific intercontinental discontinuity. Ann Mo Bot Gard..

[61] Wen J, Nowicke JW (1999). Pollen ultrastructure of *Panax* (the ginseng genus, Araliaceae), an eastern Asian and eastern North American disjunct genus. Am J Bot.

[62] Zozomová-Lihová J, Marhold K, Španiel S (2014). Taxonomy and evolutionary history of *Alyssum montanum* (Brassicaceae) and related taxa in southwestern Europe and Morocco: Diversification driven by polyploidy, geographic and ecological isolation. Taxon.

[63] Otto SP (2007). The evolutionary consequences of polyploidy. Cell.

[64] Ohno S (1970). The enormous diversity in genome sizes of fish as a reflection of nature’s extensive experiments with gene duplication. Trans Am Fish Soc.

[65] Force A, Lynch M, Pickett FB, Amores A, Yan Y, Postlethwait J (1999). Preservation of duplicate genes by complementary, degenerative mutations. Genetics.

[66] Wendel JF (2000). Genome evolution in polyploids. Plant Mol Biol.

[67] Comai L (2005). The advantages and disadvantages of being polyploid. Nat Rev Genet.

[68] Senchina DS, Alvarez I, Cronn RC, Liu B, Rong J, Noyes RD (2003). Rate variation among nuclear genes and the age of polyploidy in Gossypium. Mol Biol Evol.

[69] Lynch M, Conery JS (2000). The evolutionary fate and consequences of duplicate genes. Science.

[70] Lin J-Y, Stupar RM, Hans C, Hyten DL, Jackson SA (2010). Structural and functional divergence of a 1-Mb duplicated region in the soybean (*Glycine max*) genome and comparison to an orthologous region from *Phaseolus vulgaris*. Plant Cell.

[71] Wang S, Adams KL (2015). Duplicate gene divergence by changes in microRNA binding sites in *Arabidopsis* and *Brassica*. Genome Biol Evol.

[72] Akhunov ED, Sehgal S, Liang H, Wang S, Akhunova AR, Kaur G (2013). Comparative analysis of syntenic genes in grass genomes reveals accelerated rates of gene structure and coding sequence evolution in polyploid wheat. Plant Physiol.

[73] Bailey C (2003). Characterization of angiosperm nrDNA polymorphism, paralogy, and pseudogenes. Mol Phylogen Evol.

[74] Nieto Feliner G, Rossello JA (2007). Better the devil you know? Guidelines for insightful utilization of nrDNA ITS in species-level evolutionary studies in plants. Mol Phylogenet Evol.

[75] Baldwin BG, Sanderson MJ, Wojciechowski MF, Campbell CS, Donoghue MJ (1995). The ITS region of nuclear ribosomal DNA: A valuable source of evidence on angiosperm phylogeny. Ann Missouri Bot Gard.

[76] Zimmer EA, Wen J (2013). Reprint of: using nuclear gene data for plant phylogenetics: progress and prospects. Mol Phylogenet Evol.

[77] Li MR, Wang XF, Zhang C, Wang HY, Shi FX, Xiao HX (2013). A simple strategy for development of single nucleotide polymorphisms from non-model species and its application in *Panax*. Int J Mol Sci.

[78] Li MR, Shi FX, Zhou YX, Li YL, Wang XF, Zhang C (2015). Genetic and epigenetic diversities shed light on the domestication of cultivated ginseng (*Panax ginseng*). Mol Plant.

[79] Thompson JD, Gibson TJ, Plewniak F, Jeanmougin F, Higgins DG (1997). The CLUSTAL_X windows interface: flexible strategies for multiple sequence alignment aided by quality analysis tools. Nucleic Acids Res.

[80] Hall TA (1999). BioEdit: a user-friendly biological sequence alignment editor and analysis program for Windows 95/98/NT.

[81] Ronquist F, Teslenko M, van der Mark P, Ayres DL, Darling A, Hohna S (2012). MrBayes 3.2: efficient Bayesian phylogenetic inference and model choice across a large model space. Syst Biol.

[82] Darriba D, Taboada GL, Doallo R, Posada D (2012). jModelTest 2: more models, new heuristics and parallel computing. Nat Methods.

[83] Yang Z (2007). PAML 4: phylogenetic analysis by maximum likelihood. Mol Biol Evol.

[84] Xu B, Yang Z (2013). PAMLX: a graphical user interface for PAML. Mol Biol Evol.

[85] Schmieder R, Edwards R (2011). Quality control and preprocessing of metagenomic datasets. Bioinformatics.

[86] Li H, Durbin R (2010). Fast and accurate long-read alignment with Burrows-Wheeler transform. Bioinformatics.

[87] Li H, Handsaker B, Wysoker A, Fennell T, Ruan J, Homer N (2009). Genome project data processing S. The sequence alignment/Map format and SAMtools. Bioinformatics.

[88] Librado P, Rozas J (2009). DnaSP v5: a software for comprehensive analysis of DNA polymorphism data. Bioinformatics.

[89] Tajima F (1983). Evolutionary relationship of DNA sequences in finite populations. Genetics.

