# Supplementary material for: The impacts of polyploidy, geographic and ecological isolations on the diversification of Panax (Araliaceae)
Source: BMC Plant Biol. 2015 Dec 21;15:297. doi: 10.1186/s12870-015-0669-0 (PMC4687065; doi:10.1186/s12870-015-0669-0)
Supplement: Additional file 1: Table S1. — Accessions of Panax species and outgroups sampled from the 36 single copy nuclear genes. (DOCX 43 kb) [file 12870_2015_669_MOESM1_ESM.docx]

Table S1. Accessions of *Panax* species and outgroups sampled from the 36 single copy nuclear genes.

| Species | Geographic location | Sample size |
| --- | --- | --- |
| *Panax ginseng* | Jilin, China | 15 |
| *Panax quinquefolius* | Wisconsin, USA | 13 |
|  | Jilin, China | 7 |
| *Panax notoginseng* | Yunnan, China | 11 |
| *Panax bipinnatifidus* species complex | Yunnan, China  Sichuan, China | 4 |
| *Panax stipuleanatus* | Yunnan, China | 8 |
| *Aralia chinensis* | Jilin, China | 4 |
