# Supplementary material for: The impacts of polyploidy, geographic and ecological isolations on the diversification of Panax (Araliaceae)
Source: BMC Plant Biol. 2015 Dec 21;15:297. doi: 10.1186/s12870-015-0669-0 (PMC4687065; doi:10.1186/s12870-015-0669-0)
Supplement: Additional file 2: Table S2. — Detailed information of the chloroplast and nuclear genes used in this study. (DOCX 58 kb) [file 12870_2015_669_MOESM2_ESM.docx]

Table S2. Detailed information of the chloroplast and nuclear genes used in this study.

| Gene | Length (bp) | Segregating sites^$^ | Sample size^&^ | Model | Ta (°C) | Source^#^ |
| --- | --- | --- | --- | --- | --- | --- |
| chloroplast genome | 144,303 | 11,506 | 12 | GTR+G | NA | NCBI |
| combined cpDNA* | 3,031 | 211 | 11 | TVM+G | NA | NCBI |
| *ITS* | 615 | 157 | 244 | TrN+G | NA | NCBI |
| *PGN7* | 696 | 93 | 88 | TPM3uf+G | 54 | This study |
| *Z7* | 378 | 57 | 59 | TPM1uf+G | 52 | This study |
| *Z8* | 586 | 48 | 63 | HKY+G | 54 | This study |
| *Z14* | 864 | 86 | 51 | TPM1uf+G | 54 | This study |
| *Z15* | 743 | 106 | 54 | TIM2+G | 50 | This study |
| *W16* | 396 | 121 | 51 | TrN+G | 52 | This study |
| *W28* | 330 | 35 | 38 | HKY+G | 60 | This study |

*, the combined cpDNA contains the four chloroplast genes *trnD*, *psbK-psbI,* *rbcL* and *ycf1*;

#, the sequences of nrITS and chloroplast genome were downloaded from NCBI;

&, numbers of haplotype obtained from the dataset of chloroplast and nuclear genes.
