# Supplementary material for: The impacts of polyploidy, geographic and ecological isolations on the diversification of Panax (Araliaceae)
Source: BMC Plant Biol. 2015 Dec 21;15:297. doi: 10.1186/s12870-015-0669-0 (PMC4687065; doi:10.1186/s12870-015-0669-0)
Supplement: Additional file 3: Table S3. — Accession numbers of the Panax species and outgroups used in this study. (DOCX 23 kb) [file 12870_2015_669_MOESM3_ESM.docx]

Table S3. Accession numbers of the *Panax* species and outgroups used in this study.

| Species | *trnD* | *psbI* | *rbcL* | *Ycf2* | *ITS* |
| --- | --- | --- | --- | --- | --- |
| *Aralia chinensis* |  |  |  |  | AF242256 |
| *Aralia undulata* | KC456163 | KC456163 | KC456163 | KC456163 |  |
| *Panax trifolius* | HQ112704 | HQ112799 | HQ112614 | KM210224 | HQ112445, HQ112446, U41697, U41698 |
| *Panax stipuleanatus* | HQ112699 | HQ112795 | HQ112601 | KM210223 | U41695, HQ112441, AY271922,  HQ112444, HQ112442, U41696,  AY271921, JX680330, HQ112443 |
| *Panax pseudoginseng* | AY275918 | HQ112791 | HQ112615 | KM210218 | U41693, AY389046, AY233327,  U41694, HQ112437, HQ112438 |
| *Panax japonicus* | HQ112685 | HQ112778 | KM210146 | KM210212 | FJ980423, HQ112428, HQ112411,  HQ112426, HQ112432, HQ112431,  FJ980424, HQ112425, AF263373,  HQ112427, HQ112430, AB569092  HQ112410, AY271918, HQ112433, HQ112429 |
| *Panax quinquefolius* | HQ112697 | JX896434 | U50250 | KM210220 | FJ606755, HQ112440, HQ112439,  HM446506, U41689, U41688, U41687 |
| *Panax ginseng* | KF431956 | KC686332 | KM210144 | KM210210 | U41681, KF727967, DQ284918,  U41682, AJ786235, HM446498,  HM446500, KF727964, EU592027,  HM446502, U41680, HQ112416,  AF274533, DQ339099, KF727965,  KF727970, HM446499, AY233326,  HQ112415, AB043872, DQ339098,  KF727966, KF727971, AF274534,  EU592021, FJ593178, KF727974,  DQ339097, KF727968, KF727972,  AF274532, EU592029, AY548192, HM446504, KF727969, KF727973,  AB043871, EU592025, HM446503,  HM446501 |
| *Panax notoginseng* | HQ112693 | KJ566590 | KJ566590 | KM210217 | JQ764993, HQ112436, HQ112434,  U41685, JQ764992, AY271919,  HQ112435, JX680329, U41684  JQ764991 |
| *Panax bipinnatifidus* species complex | HQ112636  HQ112633  HQ112671 | HQ112735  HQ112800  HQ112719 | KM210139  KM210138  KM210142 | KM210208  KM210206  KM210205 | HQ112374, HQ588762, HQ112451,  AY725136, HQ112456, HQ112417,  AY233323, HQ112423, FJ872552,  HQ112450, HQ141404, U41700,  HQ112373, HQ112424, HQ112422  HQ588769, FJ853614, U41678,  HQ112368, HQ112372, HQ112418,  HQ112421, FJ872548, FJ853619,  U41686, HQ112369, AY233328,  U41683, HQ112420, HQ588768,  FJ853618, U41692, HQ112387,  HQ112398, AH010327, HQ112419,  HQ588770, HQ141398, HQ112363,  HQ112455, HQ112400, U41679,  HQ112391, FJ853613, FJ872555,  HQ112365, HQ112395, HQ112399,  HQ112408, HQ112390, HQ588766,  FJ872547, HQ112364, HQ112406,  HQ112404, HQ112410, HQ112380,  FJ872550, AY233320, HQ112413,  AY271923, HQ112405, HQ112409,  HQ112379, HQ588771, HQ141400,  HQ112414, AY233331, AY233325,  U41703, HQ112378, HQ588767,  HQ141399, HQ112389, AY233329,  AY233324, AF263376, HQ112377,  FJ872551, FJ872553, HQ112412,  HQ112403, HQ112370, HQ112407,  HQ112376, HQ588772, FJ853615,  HQ112411, AY233330, HQ112362,  AY271924, HQ112375, HQ588774,  HQ141402, HQ112388, FJ872554,  HQ112392, AY271920, FJ872546,  HQ588773, FJ872556, HQ112381,  HQ112382, HQ112393, HQ112402,  HQ588765, HQ112367, HQ141403,  U41690, HQ112383, HQ112394,  HQ112401, FJ853616, HQ112454,  AY233322, HQ112366, HQ112385,  HQ112447, HQ112397, HQ588763,  HQ112453, AY233321, U41691,  HQ112386, HQ112449, HQ112396,  HQ588764, HQ112452, AY725135,  U41699, HQ112371, HQ112448,  HQ112384 |
