# Supplementary material for: The impacts of polyploidy, geographic and ecological isolations on the diversification of Panax (Araliaceae)
Source: BMC Plant Biol. 2015 Dec 21;15:297. doi: 10.1186/s12870-015-0669-0 (PMC4687065; doi:10.1186/s12870-015-0669-0)
Supplement: Additional file 4: Table S4. — Nucleotide variation patterns of the 36 single copy nuclear genes used in this study. (DOCX 34 kb) [file 12870_2015_669_MOESM4_ESM.docx]

Table S4. Nucleotide variation patterns of the 36 single copy nuclear genes used in this study.

| **Locus** | ***Panax ginseng^&^*** | | | | | | | |  | ***Panax notoginseng*** | | | | | | | |  | ***Panax* *quinquefolius*** | | | | | | | |
| --- | --- | --- | --- | --- | --- | --- | --- | --- | --- | --- | --- | --- | --- | --- | --- | --- | --- | --- | --- | --- | --- | --- | --- | --- | --- | --- |
|  | **Ss*** | **St^#^** | **πt** | **πsyn** | **Ssyn** | **πnon** | **Snon** | **ka/ks** |  | **Ss** | **St** | **πt** | **πsyn** | **Ssyn** | **πnon** | **Snon** | **ka/ks** |  | **Ss** | **St** | **πt** | **πsyn** | **Ssyn** | **πnon** | **Snon** | **ka/ks** |
| *W3* | 20 | 30 | 0.00767 | 0.02573 | 9 | 0.00648 | 3 | 0.248 |  | 13 | 11 | 0.00328 | 0 | 0 | 0 | 0 | 0 |  | 7 | 16 | 0.00433 | 0.00076 | 1 | 0.00025 | 1 | 0.331 |
| *W6* | 8 | 23 | 0.02105 | 0.02968 | 7 | 0.00938 | 7 | 0.312 |  | 3 | 7 | 0.00783 | 0.01694 | 2 | 0.00424 | 3 | 0.248 |  | 13 | 29 | 0.02343 | 0.04282 | 11 | 0.00604 | 7 | 0.137 |
| *W7* | 19 | 47 | 0.01304 | 0.02241 | 9 | 0.00408 | 3 | 0.18 |  | 6 | 9 | 0.00344 | 0.02045 | 3 | 0.00214 | 1 | 0.104 |  | 11 | 37 | 0.01144 | 0.02116 | 8 | 0.00496 | 4 | 0.232 |
| *W13* | 6 | 15 | 0.00967 | 0.01222 | 1 | 0.0033 | 3 | 0.269 |  | 2 | 0 | 0 | 0 | 0 | 0 | 0 | 0 |  | 7 | 16 | 0.01118 | 0.01266 | 2 | 0.00415 | 1 | 0.326 |
| *W22* | 41 | 48 | 0.01921 | 0.03897 | 3 | 0.00982 | 5 | 0.247 |  | 5 | 6 | 0.00399 | 0 | 0 | 0.00292 | 2 | 0 |  | 4 | 14 | 0.01023 | 0.01723 | 3 | 0.00668 | 3 | 0.385 |
| *W26* | 9 | 22 | 0.00949 | 0.02096 | 5 | 0.00416 | 6 | 0.196 |  | 4 | 9 | 0.00148 | 0.00542 | 4 | 0.00036 | 1 | 0.067 |  | 5 | 20 | 0.0086 | 0.02053 | 5 | 0.00445 | 4 | 0.214 |
| *W28* | 4 | 5 | 0.00141 | 0 | 0 | 0 | 0 | 0 |  | 4 | 6 | 0.00303 | 0 | 0 | 0.00339 | 3 | 0 |  | 2 | 4 | 0.00188 | 0 | 0 | 0.00138 | 1 | 0 |
| *W31* | 5 | 5 | 0.00266 | 0 | 0 | 0.00055 | 2 | 0 |  | 1 | 0 | 0 | 0 | 0 | 0 | 0 | 0 |  | 2 | 2 | 0.00026 | 0.00123 | 1 | 0.00021 | 1 | 0.167 |
| *W32* | 41 | 44 | 0.00471 | NA | NA | NA | NA | NA |  | 7 | 8 | 0.00459 | NA | NA | NA | NA | NA |  | 16 | 17 | 0.0102 | NA | NA | NA | NA | NA |
| *W35* | NA | | | | | | | |  |  | 5 | 0.00314 | 0.01869 | 5 | 0 | 0 | 0 |  | 0 | 23 | 0.01408 | 0.05151 | 15 | 0.00285 | 4 | 0.054 |
| *W39* | 18 | 31 | 0.00957 | 0.02414 | 1 | 0.00416 | 1 | 0.17 |  | 4 | 5 | 0.00119 | 0 | 0 | 0 | 0 | 0 |  | 27 | 33 | 0.01042 | 0.02635 | 1 | 0 | 0 | 0 |
| *W47* | 9 | 25 | 0.00985 | 0.01862 | 4 | 0.00432 | 4 | 0.23 |  | 7 | 22 | 0.01397 | 0 | 0 | 0 | 0 | 0 |  | 9 | 24 | 0.01155 | 0.0178 | 4 | 0.00382 | 4 | 0.212 |
| *W48* | NA | | | | | | | |  |  | 58 | 0.04566 | NA | NA | NA | NA | NA |  |  | 35 | 0.04155 | NA | NA | NA | NA | NA |
| *W53* |  | 14 | 0.00388 | 0.0039 | 2 | 0.00823 | 6 | 2.114 |  | 0 | 1 | 0.00037 | 0 | 0 | 0 | 0 | 0 |  | 8 | 10 | 0.00118 | 0.00099 | 1 | 0.00155 | 3 | 1.572 |
| *W54* | 11 | 10 | 0.00309 | 0.00223 | 3 | 0.00339 | 7 |  |  | NA | | | | | | | |  | NA | | | | | | | |
| *W59* | 7 | 10 | 0.00539 | 0.00056 | 1 | 0.00363 | 4 | 6.549 |  | 2 | 0 | 0 | 0 | 0 | 0 | 0 | 0 |  | 0 | 5 | 0.00525 | 0 | 0 | 0.00264 | 2 | 0 |
| *W60* | 18 | 23 | 0.01352 | NA | NA | NA | NA | NA |  | 2 | 5 | 0.00222 | NA | NA | NA | NA | NA |  | 17 | 23 | 0.0133 | NA | NA | NA | NA | NA |
| *W65* | NA | | | | | | | |  |  | 1 | 0.00021 | 0 | 0 | 0.00165 | 1 | 0 |  |  | 41 | 0.00397 | 0.01124 | 2 | 0.00361 | 3 | 0.391 |
| *W76* | 17 | 38 | 0.00729 | 0.01238 | 4 | 0.00733 | 9 | 0.59 |  | 11 | 9 | 0.00168 | 0.00255 | 1 | 0.00157 | 1 | 0.613 |  | 24 | 44 | 0.00981 | 0.01294 | 4 | 0.00999 | 6 | 0.768 |
| *W79* | 7 | 14 | 0.01091 | 0.02063 | 4 | 0.01276 | 5 | 0.615 |  | 5 | 7 | 0.00193 | 0.00674 | 4 | 0.00186 | 3 | 0.275 |  | 2 | 10 | 0.00893 | 0.01892 | 4 | 0.00986 | 3 | 0.517 |
| *W80* | 10 | 11 | 0.00178 | 0.00747 | 3 | 0.00012 | 1 | 0.016 |  | 8 | 4 | 0.00324 | 0.00276 | 1 | 0 | 0 | 0 |  | 3 | 6 | 0.0038 | 0.00599 | 1 | 0.00189 | 2 | 0.314 |
| *W83* | 15 | 61 | 0.01052 | 0.03293 | 11 | 0.00266 | 7 | 0.079 |  | 12 | 47 | 0.01285 | 0.02424 | 6 | 0.00458 | 4 | 0.187 |  | 14 | 61 | 0.01321 | 0.03409 | 14 | 0.00408 | 6 | 0.117 |
| *W85* | 13 | 20 | 0.00726 | 0.03723 | 4 | 0.0037 | 7 | 0.097 |  | 6 | 3 | 0.00325 | 0.01599 | 1 | 0 | 0 | 0 |  | 8 | 18 | 0.01328 | 0.04849 | 6 | 0.00328 | 1 | 0.066 |
| *Z8* | 17 | 27 | 0.00872 | 0.02501 | 8 | 0.003 | 5 | 0.118 |  | 12 | 4 | 0.00112 | 0 | 0 | 0.00061 | 1 | 0 |  | 2 | 13 | 0.00681 | 0.01749 | 2 | 0 | 0 | 0 |
| *Z15* | 9 | 9 | 0.00066 | 0.00924 | 2 | 0.00174 | 6 | 0.158 |  | 10 | 5 | 0.00176 | 0 | 0 | 0 | 0 | 0 |  | 5 | 12 | 0.00269 | 0 | 0 | 0.00045 | 2 | 0 |
| *Z20* | 6 | 11 | 0.01236 | 0.02177 | 2 | 0.0072 | 5 | 0.328 |  | 2 | 1 | 0.00186 | 0.01096 | 1 | 0 | 0 | 0 |  | 7 | 11 | 0.00851 | 0.02119 | 3 | 0 | 0 | 0 |
| *Z24* | 21 | 38 | 0.01028 | 0.00101 | 1 | 0 | 0 | 0 |  | 24 | 26 | 0.01102 | 0.01391 | 1 | 0.00912 | 2 | 0.654 |  | 17 | 30 | 0.0108 | 0.01527 | 1 | 0.00474 | 1 | 0.308 |
| *Z26* | 18 | 20 | 0.00944 | 0.02907 | 2 | 0 | 0 | 0 |  | 2 | 3 | 0.0021 | 0 | 0 | 0 | 0 | 0 |  | 5 | 22 | 0.00698 | 0.03847 | 3 | 0.00108 | 3 | 0.027 |
| *Z35* | 16 | 46 | 0.02937 | 0.01633 | 3 | 0.02324 | 15 | 1.43 |  | 5 | 13 | 0.00447 | 0.00701 | 1 | 0.00227 | 3 | 0.323 |  | 4 | 37 | 0.01907 | 0.01553 | 4 | 0.01004 | 10 | 0.643 |
| *Z43* | 10 | 27 | 0.01406 | 0 | 0 | 0.00082 | 2 | 0 |  | 7 | 4 | 0.00068 | 0.00588 | 2 | 0 | 0 | 0 |  | 3 | 21 | 0.01414 | 0.01165 | 1 | 0 | 0 | 0 |
| *Z46* | 6 | 13 | 0.01108 | 0.04765 | 7 | 0.00183 | 4 | 0.037 |  | 1 | 8 | 0.00386 | 0.01463 | 5 | 0 | 0 | 0 |  | 5 | 13 | 0.00718 | 0.03047 | 7 | 0.00246 | 3 | 0.079 |
| *Z58* | 24 | 26 | 0.00208 | 0.00259 | 4 | 0.00363 | 16 | 1.404 |  | 17 | 5 | 0.00189 | 0 | 0 | 0.00198 | 1 | 0 |  | 5 | 7 | 0.00029 | 0.00241 | 4 | 0.0006 | 3 | 0.25 |
| *Z63* | 49 | 92 | 0.02536 | 0.02303 | 6 | 0.02128 | 14 | 0.923 |  | 8 | 16 | 0.00378 | 0.01633 | 3 | 0.00386 | 3 | 0.235 |  | 55 | 94 | 0.02553 | 0.03906 | 10 | 0.00876 | 11 | 0.22 |
| *Z64* | 18 | 43 | 0.00725 | 0.01783 | 18 | 0.00088 | 3 | 0.049 |  | 7 | 18 | 0.00333 | 0.01409 | 9 | 0 | 0 | 0 |  | 9 | 35 | 0.00771 | 0.01846 | 14 | 0.00173 | 1 | 0.093 |
| *Z69* | 12 | 28 | 0.00972 | 0.02064 | 8 | 0.00433 | 10 | 0.207 |  | 9 | 11 | 0.0031 | 0.00388 | 3 | 0 | 0 | 0 |  | 14 | 31 | 0.01256 | 0.02954 | 10 | 0.0026 | 4 | 0.086 |
| *Z70* | 11 | 24 | 0.0096 | 0.00944 | 2 | 0.00466 | 3 | 0.492 |  | 7 | 3 | 0.00147 | 0 | 0 | 0.00118 | 1 | 0 |  | 3 | 18 | 0.01076 | 0.00868 | 1 | 0.00325 | 5 | 0.373 |

NA, no available information; *, species-specific SNPs are calculated based on the total segregating sites of the three species; #, the SNPs that showed monomorphic within each species were not included;&, nucleotide diversity of *Panax ginseng* was retrieved from our previous study (Li et al., 2015).
