# Supplementary material for: The impacts of polyploidy, geographic and ecological isolations on the diversification of Panax (Araliaceae)
Source: BMC Plant Biol. 2015 Dec 21;15:297. doi: 10.1186/s12870-015-0669-0 (PMC4687065; doi:10.1186/s12870-015-0669-0)
Supplement: Additional file 5: Table S5. — Detailed information of the 36 single copy nuclear genes used in this study. (DOCX 22 kb) [file 12870_2015_669_MOESM5_ESM.docx]

Table S5. Detailed information of the 36 single copy nuclear genes used in this study.

| Primer Name | Primer sequences (5'-3') | Product  size（bp） | SNP* | Annealing temperature（℃） | Source |
| --- | --- | --- | --- | --- | --- |
| *W3* | F: TTACAAGGCTGGGATGACTC  R: ATATGCCACCACTCCAACAA | 768 | 51 | 54 | Li et al. 2013, 2015 |
| *W6* | F: CTGGTGCTCGGGTTCTGTAT  R: GGCTGAGCGACATCAGGAAA | 329 | 39 | 54 | Li et al. 2013, 2015 |
| *W7* | F: TGTTCCCGCTAGGTACAACT  R: CAACACCCTTTCCAGGCTTA | 814 | 63 | 56 | Li et al. 2013, 2015 |
| *W13* | F: TGAAGAAGTCTGCCAAACCG  R: TGTCACCACCTCCAAACTCG | 622 | 25 | 50 | Li et al. 2013, 2015 |
| *W22* | F: GTGCCGTTGAGTATGAAGAC  R: AGCTGCCTGAAGAAGTGCTA | 363 | 58 | 48 | Li et al. 2013, 2015 |
| *W26* | F: AAGAAGGGTAGCAAGAAAGG  R: CGTCAAGAATCCCAAGTCCA | 760 | 33 | 56 | Li et al. 2013, 2015 |
| *W28* | F: GGGGTGGGAATTTGGAAGTA  R: TGAAGGAGCATCGGAACCAT | 421 | 12 | 48 | Li et al. 2013, 2015 |
| *W31* | F: TGTGAGCAAGGGATGAGGTT  R: AAGTGCAGATCACCCGAAGA | 284 | 8 | 52 | Li et al. 2013, 2015 |
| *W32* | F: TTCTGACCCACCCAATCTTC  R: AGCCGCCTTTCGTAACTCTT | 332 | 65 | 46 | Li et al. 2013, 2015 |
| *W35* | F: TCATCATTGACTCCACCACT  R: GAGATGGGGACAAAAGCAAT | 326 | 24 | 49 | Li et al. 2013, 2015 |
| *W39* | F: ACCACCAGTAGTACGATTCG  R: CAGGGGAGTTTGGGTTTGGA | 1033 | 61 | 51 | Li et al. 2013, 2015 |
| *W47* | F: CGGAAGGTCGCTTTTACTCG  R: CAGGCTAATTTGCCCGCACT | 798 | 40 | 53 | Li et al. 2013, 2015 |
| *W48* | F: CTGGTGCTCGGGTTCTGTAT  R: CTAGGCTTGAATTGATCTGC | 340 | 102 | 48 | Li et al. 2013, 2015 |
| *W53* | F: GGATTCATGCTGAGAAGACC  R: GCACCTGGAAGAAGGATGTC | 335 | 20 | 53 | Li et al. 2013, 2015 |
| *W54* | F: CCCCTAATCGAACTGTTTGG  R: CACTACTCTTCTCCGCATCA | 231 | 10 | 52 | Li et al. 2013, 2015 |
| *W59* | F: AACATTGCCGATAACCCCAC  R: CTGAGAGCACCAAAGAAATC | 378 | 13 | 52 | Li et al. 2013, 2015 |
| *W60* | F: CCAGTGTCCAACAACAATAG  R: GGAGGCAAATGTCGGTGAAA | 613 | 53 | 52 | Li et al. 2013, 2015 |
| *W65* | F: CAGAGGTAGTGAGCTTCTCA  R: AGCAGCAATAGTTAGCAGCC | 1127 | 85 | 52 | Li et al. 2013, 2015 |
| *W76* | F: GATGCTTTGATGCCTTCTGG  R: CGTCTTTATCAGCGTCCTCT | 985 | 72 | 54 | Li et al. 2013, 2015 |
| *W79* | F: CTCGCTTCTCACCAAGTCAG  R: ATGTCCTCCCCTTTGTTCTG | 453 | 22 | 51 | Li et al. 2013, 2015 |
| *W80* | F: TGCCAGTTATCAGGGCTCAA  R: TTTCCTTTCCTTCGCACTCC | 540 | 24 | 50 | Li et al. 2013, 2015 |
| *W83* | F: GTAGCAGAGGAGATGGGAAT  R: GAATCGTCAAACACAAAGGG | 928 | 89 | 53 | Li et al. 2013, 2015 |
| *W85* | F: TGATGTGGAGAAGAGGAAGC  R: GCATAACACCGTCTGGCAGT | 397 | 35 | 53 | Li et al. 2013, 2015 |
| *Z8* | F: GGGAAGGAAAAGTTGCTCTG  R: TATTCGTGTTGGGGCATCTG | 802 | 41 | 56 | Li et al. 2013, 2015 |
| *Z15* | F: TGAACAGGCATTATTACTCG  R: ACTCATCCTCCTCTTGAACG | 759 | 31 | 45 | Li et al. 2013, 2015 |
| *Z20* | F: GCAAAGAAAGGGGCAAATAC  R: TCACCACTGATCCCAACAAC | 287 | 20 | 50 | Li et al. 2013, 2015 |
| *Z24* | F: GCAAAGAAAGGGGCAAATAC  R: TCACCACTGATCCCAACAAC | 984 | 78 | 54 | Li et al. 2013, 2015 |
| *Z26* | F: CCAAAGTGTCCTTGCTGCTC  R: TATTCTCCTCTGGTAGCCTG | 662 | 43 | 49 | Li et al. 2013, 2015 |
| *Z35* | F: TCAAGGTCTTTGTGGTGTT  R: TGCTTGCTTTGCTTTCTACC | 449 | 56 | 52 | Li et al. 2013, 2015 |
| *Z43* | F: CATCAAATGGAAGCGGAGAA  R: GGATAGGGTTATCGTCGTCA | 730 | 38 | 57 | Li et al. 2013, 2015 |
| *Z46* | F: CCAAGCCCTAACAATAACGG  R: GTGTGTCATCTACAAAATCC | 449 | 20 | 50 | Li et al. 2013, 2015 |
| *Z58* | F: GCTCACGCAAGCAGTGTATG  R: CTTCGTCAGATATTCCCGTC | 766 | 49 | 47 | Li et al. 2013, 2015 |
| *Z63* | F: TGTGGGAGATGATCTGCTGG  R: TTGGCAAGACGCTCTGACCT | 659 | 149 | 48 | Li et al. 2013, 2015 |
| *Z64* | F: GTGAGGATGGTGAGGGTAAG  R: TGATGCCAGCAGATGTAGTC | 675 | 60 | 51 | Li et al. 2013, 2015 |
| *Z69* | F: GTGAGCAAGCTAAACATGGC  R: TAGGACCAGCAGCAAGGATT | 943 | 156 | 54 | Li et al. 2013, 2015 |
| *Z70* | F: CGAGGCTGAAAAAGCATACT  R: TAGAGAGGAATCATTGGTTG | 630 | 36 | 51 | Li et al. 2013, 2015 |

*, the number of SNP for each locus is calculated based on the three species.
